# Sphingosine‐1‐phosphate as a key player of insulin secretion induced by high‐density lipoprotein treatment

**DOI:** 10.14814/phy2.14786

**Published:** 2021-03-26

**Authors:** Marie‐Claude Brulhart‐Meynet, Aurélien Thomas, Jonathan Sidibé, Florian Visentin, Rodolphe Dusaulcy, Valérie Schwitzgebel, Zoltan Pataky, Jacques Philippe, Nicolas Vuilleumier, Richard W. James, Yvan Gosmain, Miguel A. Frias

**Affiliations:** ^1^ Division of Endocrinology Diabetes, Nutrition and Patient Education Department of Medicine University Hospitals of Geneva and University of Geneva Geneva Switzerland; ^2^ Unit of Toxicology University Centre of Legal Medicine Lausanne‐Geneva Switzerland; ^3^ Pediatric Endocrine and Diabetes Unit Department of Pediatrics, Gynecology and Obstetrics University Hospitals of Geneva Geneva Switzerland; ^4^ Division of Laboratory Medicine Department of Diagnostic Geneva University Hospitals Geneva Switzerland; ^5^ Department of Medicine Medical Faculty, Geneva University Geneva Switzerland

**Keywords:** glucose‐stimulated insulin secretion, high‐density lipoproteins, primary pancreatic beta cells, sphingosine‐1‐phosphate, type 2 diabetes mellitus

## Abstract

Beta cell failure is one of the most important features of type 2 diabetes mellitus (T2DM). High‐density lipoprotein (HDL) has been proposed to improve β‐cell function. However, the mechanisms involved in this process are still poorly understood. The aim of this study was to investigate the contribution of sphingosine‐1‐phosphate (S1P) in the impact of HDL treatment on insulin secretion by pancreatic β‐cells and to determine its mechanisms. Primary cultures of β‐cells isolated from rat were treated with or without HDL in the presence or absence of S1P pathway inhibitors and insulin secretion response was analyzed. The S1P content of HDL (HDL‐S1P) isolated from T2DM patients was analyzed and correlated to the HDL‐induced insulin secretion. The expression of genes involved in the biosynthesis of the insulin was also evaluated. HDL as well as S1P treatment enhanced glucose‐stimulated insulin secretion (GSIS). In HDL isolated from T2DM patients, while HDL‐S1P was strongly correlated to its pro‐secretory capacity (*r* = 0.633, *p* = 0.005), HDL‐cholesterol and apolipoprotein AI levels were not. HDL‐induced GSIS was blocked by the S1P1/3 antagonist but not by the S1P2 antagonist, and was also accompanied by increased intracellular S1P in β‐cells. We also observed that HDL improved GSIS without significant changes in expression levels of insulin biosynthesis genes. Our present study highlights the importance HDL‐S1P in GSIS in T2DM patients and demonstrates that HDL induces insulin secretion by a process involving both intra‐ and extra‐cellular sources of S1P independently of an effect on insulin biosynthesis genes.

## Graphical abstract

HDL improves insulin secretion by beta cells S1P plays a role in HDL‐induced insulin secretion HDL increases intracellular S1P content.

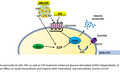

## 1 INTRODUCTION

A central feature of the etiology of type 2 diabetes mellitus (T2DM) is β‐cell failure, involving a deficit in insulin secretion and β‐cell apoptosis. Recent studies have highlighted a role for HDL in maintaining β‐cell function, by attenuating the deleterious effects of intracellular stress and limiting β‐cell apoptosis when the cells are subjected to various conditions of physiological or non‐physiological stress (Petremand et al., 2012; Roehrich et al., 2003).

There is scarce information on a more direct role of HDL in insulin secretion. Current data are controversial with some studies showing that HDL treatment can enhance glucose‐stimulated insulin secretion (GSIS) and other showing no effect on GSIS (Drew et al., 2009; Rutti et al., 2009). In patients, reconstituted HDL (rHDL: apolipoprotein AI + phospholipid complex) treatment reduced plasma glucose in T2DM by increasing plasma insulin and activating AMP‐activated protein kinase in skeletal muscle (Drew et al., 2009). Previous studies have focused on the role of its major peptide component apolipoprotein AI (apoAI) (Cochran et al., 2014; Domingo‐Espin et al., 2016; Fryirs et al., 2010). However, there is growing evidence of the importance of a lipid component of HDL, sphingosine‐1‐phosphate (S1P), to a range of beneficial effects of HDL on the vasculature (Sattler & Levkau, 2009). These include promoting endothelial integrity and angiogenesis, modulating arterial vessel tone and reducing inflammatory reactions affecting the endothelium. Plasma S1P is principally associated with HDL via apolipoprotein M (apoM) (Murata et al., 2000). Recent studies, including our own, have shown that S1P contained in the HDL particle, (defined in this manuscript by HDL‐S1P) is bioactive and has direct beneficial effects on several organs, including the heart, and notably antiapoptotic actions (Brinck et al., 2016; Levkau, 2015; Sattler et al., 2015).

Regarding the impact of the S1P content of HDL (HDL‐S1P) in T2DM, we recently showed that the concentration of HDL‐S1P is directly correlated to the capacity of HDL to protect cardiomyocytes from oxidative insult (Brinck et al., 2016). Whether HDL‐S1P can influence the impact of HDL on pancreatic β‐cells remains unknown.

The aim of this study was to analyze the effect of HDL and its constituent S1P on the insulin secretion capacity in a model of primary culture of rat β‐cell and investigate its mechanisms of action. A particular aspect was the investigation of HDL‐S1P in HDL isolated from T2DM.

## 2 MATERIAL AND METHODS

### 2.1 Participant characteristics

A total number of 18 T2DM patients were prospectively recruited at the University Hospitals of Geneva, Service of Therapeutic Education for Chronic Diseases. Ethical permission (Ethics Commission, University Hospital) was accorded for the study and informed consent was obtained from all donors. All human investigation was conducted according to the principles expressed in the Declaration of Helsinki. Participant characteristics are given in Table 1. The following clinical parameters were assessed at the University Hospital of Geneva: Glucose, Hemoglobin A1c, total cholesterol, HDL‐cholesterol and triglycerides were measured with the Cobas e501 automate using electrochemiluminescence (Roche). LDL‐cholesterol concentrations were calculated according to the Friedewald formula. A pool of plasma from healthy donors was also used to isolated HDL.

**TABLE 1:** Study cohort

	Type 2 diabetes
*n*	18
Sex (male/female)	9/9
Age, years	51 (45–67.25)
Triglycerides, mmol/L	1.385 (1.16–2.77)
Total cholesterol, mmol/L	5.05 (3.66–5.775)
LDL‐C^a^, mmol/L	2.84 (2.413–3.815) (*n* = 17)
HDL‐C, mmol/L	1.135 (0.93–1.468)
Glucose mmol/L	7 (5.775–8.725)
Hemoglobin A1c, mmol/L	7.59 (6.47–10.21)
Smokers	4/18
Coronary artery disease	2/18
Systolic blood pressure (mmHg)	131.5 (112.5–145)
Diastolic blood pressure (mmHg)	82.5 (76.25–90)
Heart rate (beat/min)	79 (75–82)
Body mass index (kg/m2)	35.75 (29.775–44.6)

Median (25–75 percentile).

^a^ Calculated by Friedewald's formula.

### 2.2 Primary Rat β‐cell sorting and primary culture

The investigation conformed to the Guide for the Care and Use of Laboratory Animals published by the US National Institutes of Health (NIH Publication No. 85–23, revised 1996) and was approved by the local ethical committee of the Geneva University Medical School. Islets of Langerhans were isolated from male rats OFA (200–250 g, 7–8 weeks old) using a standard procedure as described (Gosmain et al., 2010). β‐cells were separated from α‐cells and exocrine cells by autofluorescence‐activated sorting with the FACS Vantage SE cell sorter (Becton Dickinson) following 2 criteria: fluorescence intensities and cell size. We obtained two major populations characterized by immunohistochemistry, β‐cells, and an enriched α‐cell fraction. Fraction 1 was composed almost exclusively of insulin‐positive cells (>95%), whereas fraction 2 of a majority of glucagon‐positive cells (∼75 ± 5%) (Gosmain et al., 2012). β‐cells were cultured on extracellular matrix‐coated plates derived from 804G cells (laminin‐5‐rich extracellular matrix) in DMEM containing 10% fetal bovine serum (FBS), 11.2 mM D‐glucose, 2 mM l‐glutamine, and antibiotics. Before stimulation, β‐cells were incubated overnight with DMEM containing 1% FBS, 5.6 mM D‐glucose, 2 mM l‐glutamine, and antibiotics.

Prior to testing GSIS, β‐cells were incubated 24 h with or without HDL (400 µg/ml) in DMEM containing 0.5% fatty acid‐free bovine serum albumin (BSA), 5.6 mM D‐glucose and antibiotics (see Figure S2 for experimental protocol).

### 2.3 Measurements of insulin secretion and content

Insulin secretion was analyzed by sequential treatment with different concentrations of glucose. Briefly, β‐cells were first incubated for 2 h in depleted medium [Krebs buffer + 2.8 mM glucose + 0.1% BSA (KRB‐2.8 mM glc)] to minimize insulin secretion. Cells were then incubated for 1 h in KRB‐2.8 mM glc to determine basal secretion levels and stimulated by an additional 1 h incubation with KRB‐16.7 mM glc. In each experiment, a 2.8 mM glucose concentration is used for basal values and 16.7 mM glucose for stimulated values. Insulin cell content was measured after cell collection into a freezing acid/ethanol mixture. Insulin was quantified by an ELISA kit (High Range Rat Insulin ELISA kit Mercodia) following the manufacturer's recommendations (see Figure S1 for experimental protocol). Inhibitors or antagonists and vehicles were incubated 30 min before adding HDL. Sphingosine Kinase Inhibitor II (SK‐II, 10 µM), N,N‐Dimethylsphingosine (DMS, 10 µM), VPC23019 (2 µM, S1P1 and 3 antagonist), and JTE013 (5 µM, S1P2 antagonist) were from Cayman Chemical.

### 2.4 Preparation of HDL

For preliminary studies, HDL (*d* = 1.063–1.21 g/ml) was prepared by a standard double ultracentrifugation protocol from pooled human plasma of healthy donors as described (Brinck et al., 2016). This preparation was used in experiments if not otherwise indicated. Subsequently, and notably when individual preparations from T2DM patients were required, a more rapid isolation procedure (7 h) was developed. It entailed precipitation of apolipoprotein B containing lipoproteins by addition of sodium phosphotungstate (12 mM, pH 6.15) and MgCl_2_ (50 mM) then centrifuging (3500 g, 1 h, 4°C). The supernatant was recovered and the density adjusted to 1.21 g/L with NaBr before ultracentrifugation (5 h, 430,000 g). HDL was recovered and dialyzed against PBS‐EDTA (100 µM) or by several washes with PBS‐EDTA in microspin columns. HDL protein concentration was determined by Lowry. HDL concentration is expressed as its protein concentration.

### 2.5 RNA preparation and RT‐PCR analyses

Total RNA was isolated from primary rat β‐cells using an RNA isolation kit (Qiagen) and then reverse transcribed. Target genes involved in insulin biosynthesis and signalling pathways were analyzed by real‐time RT‐PCR using LC 480 II (Roche Diagnostics) and specific primers (see Figure S1 for experimental protocol). Target gene expression was normalized to *Rps9* and results expressed in arbitrary units (AU). The primers sequences are listed in Table S1. For *Sphk1* (Rn 00682794_g1) and *Sphk2* (Rn 01457923_g1) measurement Taqman probes (Themofisher) were used and normalized by *Gapdh* (Rn 99999916_s1).

### 2.6 HDL‐S1P measurement

S1P concentrations in HDL were determined by liquid‐chromatography tandem mass spectrometry system (LC–MS/MS). Isolated HDL was diluted 1:9 in MeOH containing 10 ng/ml internal standard (S1P‐d7, Avanti Polar Lipids Inc.) prior to injection into the LC–MS/MS (Ultimate 3000 LC Series, Thermo Fisher Scientific Inc., and 5500 QTrap^®^ triple quadrupole linear ion trap system equipped with a TurboIon Spray™ interface (AB Sciex)). Data acquisition and analysis were performed using Analyst™ software (version 1.6.2; AB Sciex) (Brinck et al., 2016). S1P concentrations in cells were determined by liquid‐chromatography tandem mass spectrometry system (LC–MS/MS) after 24 h stimulation with HDL. Samples were prepared as follow: cells were removed from medium and washed three times with cold PBS. Cells were incubated with 1.5 ml solution of ice cold methanol 80% (vol/vol) for 20 min, scraped, placed in 2 ml Eppendorf tube and centrifuged 14,000*g* for 5 min at 4°C. The supernatant was collected, transferred to a new 2 ml Eppendorf tube and lyophilized using no‐heat speed vacuum. The pellet was resuspended in 100 µl methanol for S1P quantification by LC–MS/MS.

### 2.7 S1Pd7 experiments

To evaluate the fate of S1P contained in HDL particle, we used a preparation of HDL particle that incorporated S1Pd7 (deuterium) which can be specifically identified by LC–MS/MS. We were therefore able to discriminate the S1Pd7 from the endogeneous S1P of HDL particle. S1Pd7 was incorporated in native HDL as follows; firstly S1Pd7 (methanol 95% (vol/vol)) was dried in a glass tube and incubated 45 min, 37°C with HDL particles (1 mg/ml) followed by dialysis against PBS‐EDTA (100 µM) using microspin columns. The incorporation of S1Pd7 was confirmed by LC–MS/MS using the same protocol as HDL‐S1P determination. Six different S1Pd7 concentration were evaluated (see Figure S1a for experimental protocol). The preparation with comparable amounts of S1P and S1Pd7 was chosen for the experiments (see Figure S2b and c).

To evaluate whether S1P contained in HDL particle was able to transfer into cultured β‐cells, β‐cells were incubated 24 h with or without HDL particles (400 µg/ml) that contained S1Pd7 in DMEM containing 0.5% free fatty acid bovine serum albumin (BSA), 5.6 mm D‐glucose and antibiotics (see Figure S2d for experimental protocol). At the end of the incubation, the medium was collected for analysis. Cells were washed three times with cold PBS before incubation with 1.5 ml of frozen methanol 80% (vol/vol) for 20 min, scraped, placed in 2 ml Eppendorf tube and centrifuged 14,000*g* for 5 min at 4°C. The supernatant was collected, transferred to a new 2 ml Eppendorf tube and lyophilized using no‐heat speed vacuum. The pellet was resuspended in 50 µl methanol. The amounts of S1Pd7 and S1P were analyzed in the medium and the pellet, correspoding to cell content, by LC–MS/MS.

### 2.8 Statistical analysis

Values are presented as mean ± SEM if not otherwise stated. Differences between data sets were assessed by Student's *t*‐test (paired where applicable) or two‐way ANOVA with Tukey's multiple comparison post‐test, Pearson product‐moment correlation coefficient (Pearson's r). A value of *p* < 0.05 was considered significant.

## 3 RESULTS

### 3.1 Insulin secretion induced by HDL

**FIGURE 1:**
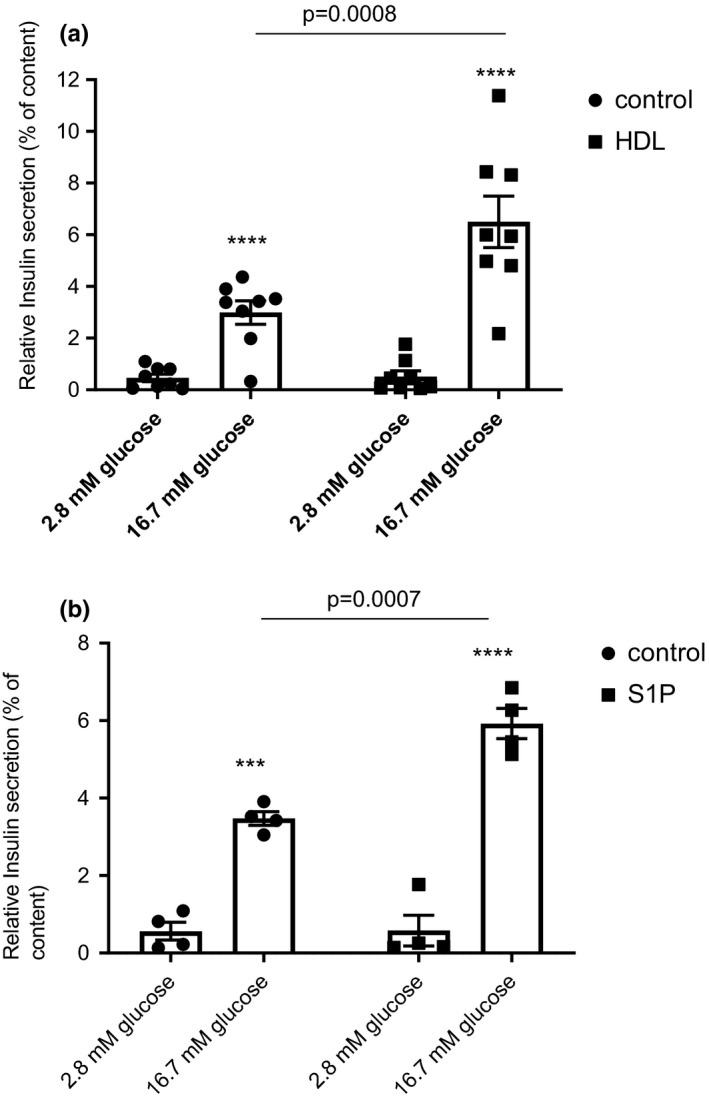
HDL and S1P treatment improves insulin secretion. HDL 400 µg/ml treatment for 24 h improved GSIS (*n* = 8) (a). S1P 400 nM treatment for 24 h improved GSIS (*n* = 4) (b). Data are mean ± SEM, statistical *p* value was calculated using a two‐way ANOVA with Tukey's multiple comparison post‐test. 2.8 mM glucose was compared to respective 16.7 mM glucose and considered significantly different with a *p* value of *<0.05, **<0.01 and ****<0.0001.

Consistent with other reports, our GSIS analysis performed on pancreatic β‐cells revealed that treatment with HDL (400 µg/ml) for 24 h doubled insulin secretion compared to non‐treated cells (control) (Figure 1a). Treatment with HDL 200 µg/ml was borderline significant (*p* = 0.06) (see Figure S3). HDL was not present during GSIS. Of note, cell insulin content was similar between the two groups; (378.5 ± 55 µg/L in control vs. 428.2 ± 103 µg/L in HDL‐treated cells, (*p* = 0.58), data not shown). The absolute amount of basal insulin secretion was not different between the two groups (2.0 ± 0.5 µg/L in control vs. 2.7 ± 1.4 µg/L, *p* = 0.80). We also confirmed, in our model, previous suggestions that S1P (400 nM) by itself can increase insulin secretion (Figure 1b).

### 3.2 S1P content in HDL correlates with the level of insulin secretion in T2DM patients

**FIGURE 2:**
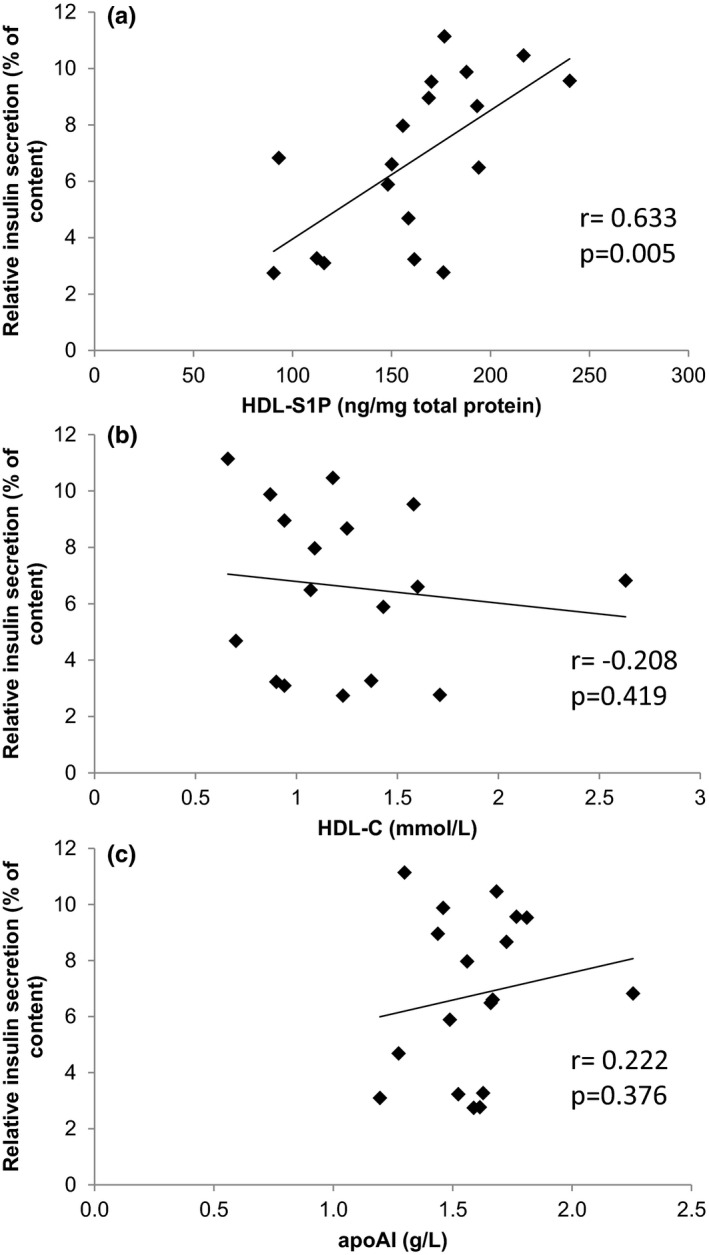
HDL S1P content from diabetic patients correlates with pro‐secretory capacity Pancreatic β‐cells were incubated with HDL (400 µg/ml) from diabetic patients (*n* = 18) for 24 h and insulin secretion was evaluated following the GSIS protocol. The effects were correlated to the HDL S1P content. (a) Correlation between Insulin secretion and HDL‐cholesterol. (b) Correlation between Insulin secretion and apoAI. (c) Analyses used the nonparametric Spearman test.

Our recent studies (Brinck et al., 2016) have highlighted the importance of the HDL‐S1P to HDL function in T2DM. We therefore analysed a potential role for S1P in HDL‐induced GSIS. To examine the relevance of S1P in HDL in our model, we analysed HDL‐induced GSIS as a function of HDL‐S1P concentration using HDL preparations from T2DM patients. The characteristics of diabetic patients are shown in Table 1. As shown in Figure 2, the level of insulin secretion was directly correlated to the amount of S1P in HDL (Figure 2a), but not to HDL‐cholesterol (HDL‐C) (Figure 2b) or apoAI (Figure 2c). Our results show for the first time that while the content of S1P reflects the GSIS potential of HDL, apoAI or HDL‐C do not.

### 3.3 S1P is required for HDL‐induced GSIS improvement

**FIGURE 3:**
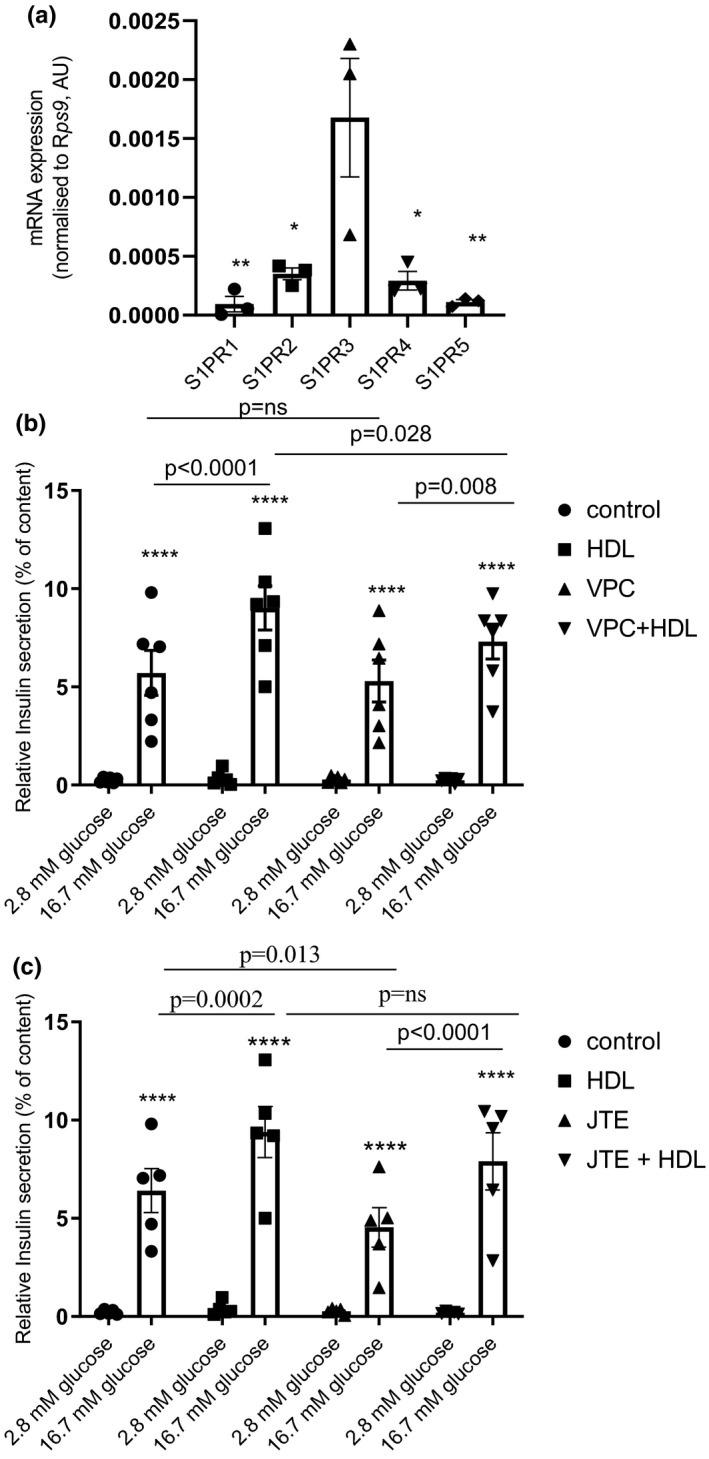
HDL treatment improves insulin secretion via S1P 1/3. RNA expression of S1P receptors subtypes quantified by qPCR, normalized by Rps9 expression and expressed in arbitrary unit (AU). S1P3 expression is increased compared to other S1P receptor subtypes. Data are mean ± SEM, statistical *p* value was calculated using a one‐way ANOVA with Tukey's multiple comparison post‐test. S1P 1/3 antagonist (VPC23019, 2 µM) (b) and S1P2 antagonist (JTE013, 5 µM) (c) were incubated 1 h before treatment with HDL 400 µg/ml. Graphs represented GSIS and data are mean ± SEM, statistical *p* value was calculated using a two‐way ANOVA with Tukey's multiple comparison post‐test. 2.8 mM glucose (basal) was compared to respective 16.7 mM glucose (stimulated) and considered significantly different with a *p* value of ****<0.0001 (*n* = 5).

The specific role of S1P was further evaluated by focusing on S1P receptor subtypes. Cells were incubated with specific antagonists 30 min before HDL treatment. The expression of S1P receptor subtypes in our model of primary beta cells measured (see Figure 3a). We focused on S1P1, S1P2, and S1P3 as they are the subtype that have been implicated in the effects of S1P. In these experiment we determined whether the impact of HDL was significantly reduced when the cells were treated with the antagonists. While incubation with the S1P1/3 antagonist (VPC23019, 2 µM) significantly reduced the pro‐secretory effect of HDL (HDL vs. VPC + HDL, *p* = 0.028), the S1P2 antagonist (JTE013, 5 µM) had no significant effect (HDL vs. JTE + HDL, *p* = ns), as HDL treatment still enhanced GSIS. Interestingly, treatment with the S1P2 antagonist alone significantly diminished insulin secretion induced by GSIS (control vs. JTE, *p* = 0.013), (Figure 3).

**FIGURE 4:**
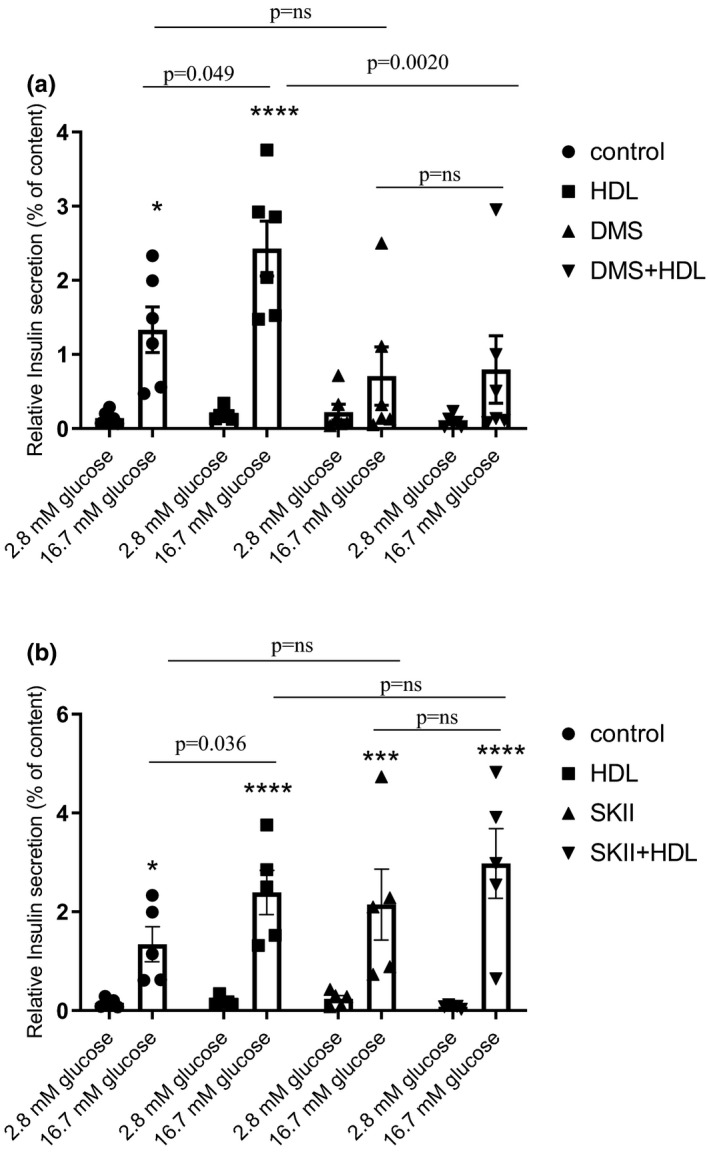
HDL treatment improves insulin secretion via Sphk2. Nonspecific Sphk inhibitor (DMS, 10 µM) (a) and Sphk1 inhibitor (SK‐II, 10 µM) (b) were incubated 30 min before treatment with or without HDL (400 µg/ml). Graphs represented GSIS and data are mean ± SEM, statistical *p* value was calculated using a two‐way ANOVA with Tukey's multiple comparison post‐test. 2.8 mM glucose (basal) was compared to respective 16.7 mM glucose (stimulated) and considered significantly different with a *p* value of * <0.05, ***<0.001 and **** <0.0001 (*n* = 6)

**FIGURE 5:**
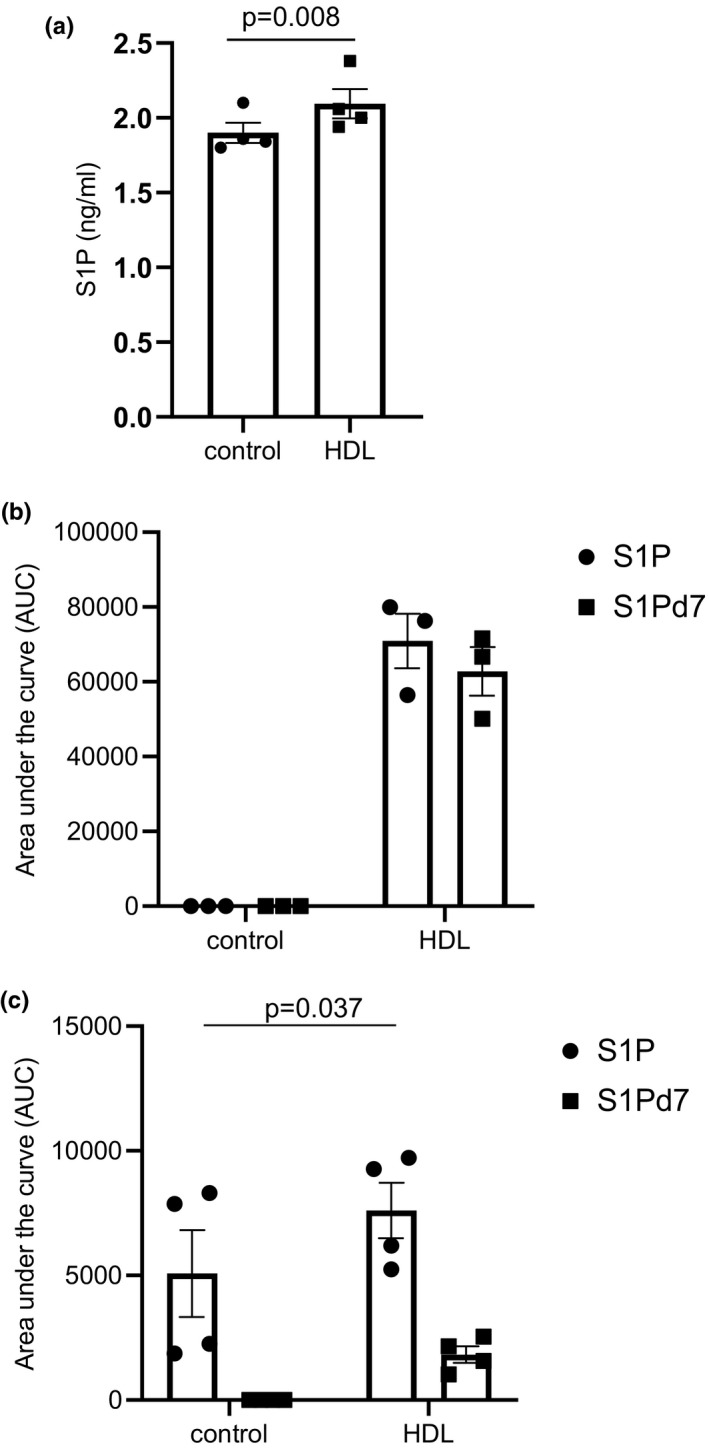
HDL transfert S1P into pancreatic β‐cells.Pancreatic β‐cellswere incubated with HDL 400 μg/ml for 24 h and S1P cellcontent was quantified by LC MS/MS (a). Data are mean ± SEM, statistical *p* value was calculated using Student‐T test paired (*n* = 4). β‐cells were treated with control medium (DMEM glucose 5.6 mmol/L, 0.5%BSA) or with control containing HDL‐S1Pd7 (400 µg/ml) (see Figure S2). After 24 h of stimulation, S1P and S1Pd7 concentrations of incubation medium (b) and cells (c) were determined by LC–MS/MS. AUC: area under the curve. Data are mean ± SEM, statistical *p* value was calculated using Student‐*T* test paired (*n* = 4)

S1P is a bioactive lipid with intra‐ and extra‐cellular effects. S1P can be generated intracellularly from sphingosine by two different isoforms of sphingosine kinase (sphk): sphk1 and sphk2. We treated cells with the sphk1 specific inhibitor, SK‐II (10 µM) or the non‐specific sphk inhibitor N,N‐dimethylsphingosine (DMS, 10 µM) 30 min before adding HDL for 24 h. The enhancement of GSIS induced by HDL treatement was significantly abolished in the presence of DMS (HDL vs. DMS + HDL, *p* = 0.020) but not SK‐II (HDL vs. SKII + HDL, *p* = ns) (Figure 4) demonstrating that inhibition of sphk1 is not sufficient for inhibiting the pro‐secretory impact of HDL treatment. This demonstrates an involvement of intracellular S1P formation by sphingosine kinase and suggests a role for sphingosine kinase 2 in this response. The expression of sphk1 and sphk2 mRNA was analysed after HDL treatment but neither was significantly changed after 24 h of treatment with HDL (see Table 2). Using LC‐MS/MS analysis, we quantified the concentration of intracellular S1P. It showed a significant increase after HDL treatment (see Figure 5a). We used S1Pd7 to elucidate the origin of intracellular S1P (see Figure S2). β‐cells were treated with control or HDL containing S1Pd7 (HDL‐S1Pd7) and the fate of S1Pd7 after 24 h of stimulation was determined (see Figure 5). As expected, while the control medium contained no S1P and no S1Pd7, the HDL‐SD1Pd7 medium contained a similar amount of S1P and S1Pd7. We also measured the amount of intracellular S1P and S1Pd7. S1P found in control cells represented the intracellular amount of S1P. In HDL‐S1Pd7‐treated cells, S1P concentration was significantly increased and S1Pd7 was found in the cells (see Figure 5). The results demonstrated that the major amount of S1Pd7 remained with HDL, but a fraction was transferred to cells. Presuming that the same amount of S1P (approximately 20%) was also transferred into the cells, this amount can explain the increase in intracellular S1P level found after HDL treatment. The experiments with sphk inhibitor and with S1Pd7 revealed (i) the important role of S1P in HDL‐induced insulin secretion as the effect of HDL is abolished by DMS and (ii) the incorporation of S1P from the HDL particle may explain the increase in intracellular levels of S1P. To our knowledge, these data demonstrate, for the first time, that HDL treatment can directly modulate intracellular S1P levels in pancreatic β‐cells.

**TABLE 2:** Gene expression

Name of gene	Control	HDL treated	*p* value
*Sphk1*	0.000017 ± 9.4E−06	0.000024 ± 7.7E−06	0.059
	100%	153.4 ± 45.8%	
*Sphk2*	0.0194 ± 0.0119	0.0209 ± 0.0108	0.352
	100%	104.6 ± 7.5%	
*MafA*	0.198 ± 0.019	0.169 ± 0.007	0.402
	100%	88.8 ± 24%	
*Pdx1*	0.273 ± 0.088	0.241 ± 0.073	0.573
	100%	91.7 ± 28.2%	
*Pax6*	0.294 ± 0.039	0.241 ± 0.008	0.336
	100%	87.7 ± 29.2%	
*Pc1*	0.132 ± 0.01	0.109 ± 0.012	0.467
	100%	78 ± 15.9%	
*Pc2*	2.766 ± 0.098	2.315 ± 0.205	0.282
	100%	90.9 ± 19.3%	
*Nkx6.1*	0.201 ± 0.017	0.176 ± 0.005	0.491
	100%	92.9 ± 34.6%	
*Tpx2*	0.012 ± 0.0003	0.0011 ± 0.0003	0.693
	100%	99.7 ± 12.4%	
*Ki67*	0.005 ± 0.0012	0.0046 ± 0.0017	0.718
	100%	90.1 ± 34.7%	
*Ddit3*	0.248 ± 0.0285	0.264 ± 0.0327	0.106
	100%	102.2 ± 3.6%	
*Bbc3*	0.0052 ± 0.0001	0.0050 ± 0.0004	0.639
	100%	106.8 ± 15.1%	
*HspA5*	0.8592 ± 0.02	0.8236 ± 0.061	0.500
	100%	86.3 ± 8.9%	
*Top2A*	0.0014 ± 0.0001	0.0013 ± 0.0001	0.794
	100%	83.6 ± 34.4%	

The expressions of target genes were evaluated 24 h after HDL treatment (400 µg/ml). No significant differences were found between non‐treated (control) and HDL treated cells.

Gene expression (arbitrary units, AU) normalized by the expression of the *Rps9* gene.

Control treated cells are considered 100% and the respective percentage ± standard deviation, statistical *p* value was calculated using Student‐*T* test.

### 3.4 Expression of insulin biosynthetic and oxidative stress genes

Several mechanisms have been proposed to explain the effect of HDL on GSIS. We analysed the expression of genes implicated in the insulin secretion process (for protocol see Figure S1b). These included expression of *Insulin*, the transcription factors *MafA*, pancreatic and duodenal homeobox 1 (*Pdx1*), paired box 6 (*Pax6*) and the enzymes proprotein convertase 1 (*Pc1*) and proprotein convertase 2 (*Pc2*). As shown in Table 2, there were no significant difference between control and HDL‐treated cells. This result suggests that HDL stimulates GSIS but not insulin biosynthesis (Table 2).

Further analyses were undertaken to evaluate the expression of genes involved in β‐cell development (Homeobox protein *Nkx*‐*6*.*1*), proliferation (microtubule nucleation factor *Tpx2*, *Antigen Ki67*) as well as oxidative stress (*Dna damage inducible transcript 3* (*Ddit3*), *Bcl2 binding component 3* (*Bbc3*), *Heat shock protein family a member 5* (*HspA5*), *Dna topoisomerase 2*‐*alpha* (*Top2A*)). The expression of all the genes remained unchanged after 24 h treatment with HDL (Table 2). Thus, the cultured β‐cells did not show evidence of stress that could impact on their capacity to secrete insulin.

## 4 DISCUSSION

The beneficial impact of HDL on pancreatic β‐cells in T2DM patients was highlighted by (Drew et al., 2009). Infusion of rHDL improved glucose levels by an increase in insulin secretion and glucose uptake by muscle (Drew et al., 2009). These results were confirmed in clinical and experimental models, where rHDL, apoAI, and HDL treatment increased insulin secretion and decreased circulating glucose (Drew et al., 2009; Rye et al., 2016; Siebel et al., 2015). The authors concluded that effects of HDL on pancreatic β‐cells were principally explained by a protective impact on cell survival against stress‐induced apoptosis, as promoted by high glucose concentrations and cytokines (Petremand et al., 2009, 2012; Rutti et al., 2009). A direct role for HDL in insulin secretion has been poorly explored although some studies propose a role for apoAI (Rye et al., 2016). We observed no correlation between circulating apoAI and insulin secretion, contrary to our observation with HDL‐S1P. It should be noted with respect to infusion studies that rHDL can rapidly assimulate S1P (Brinck et al., 2018) that may alter the composition of rHDL in such studies.

S1P has already been implicated in several actions attributed to HDL. In a previous study, we showed that HDL‐S1P content was associated with the cardioprotective capacity of HDL (Brinck et al., 2016). In the current study, we found that the insulin pro‐secretory capacity of HDL was directly associated with the HDL‐S1P content in T2DM patients. Interestingly, low HDL‐S1P has been linked to increased incidence of coronary artery disease and T2DM (Barter et al., 2007; Besler et al., 2011; Brinck et al., 2016; deGoma et al., 2008; Kontush & Chapman, 2006; Levkau, 2015). So far, the direct role of S1P in insulin secretion has not been investigated in detail.

This study implicates for the first time both extracellular and intracellular S1P in HDL‐induced insulin secretion from pancreatic β‐cells. HDL‐induced secretion is reduced (i) at the extracellular level by blocking S1P receptors (ii) and at the intracellular level by blocking S1P synthesis. In the latter context, HDL was shown to increase β‐cell S1P levels, mainly via a transfer from the HDL particle. It implies a role for an HDL‐mediated increased intracellular S1P in the insulin secretory process.

Limited data have suggest that intracellular S1P content plays a role in GSIS in cultured β‐cells with sphingosine kinase 2 being involved in this process (Cantrell Stanford et al., 2012; Ng et al., 2017; Shimizu et al., 2000). Our results concur with these suggestions. In addition, using S1Pd7, we were able to highlight the transfer of S1P from HDL particle into β‐cells. We conclude that the origin of the intracellular S1P could be the uptake from HDL particles. While further investigations are needed to clarify the specific pathway involved in HDL‐induced insulin secretion, our results suggest that intracellular S1P levels might play a role. This could be via an increase in intracellular Ca^2+^ levels, since the calcium influx rate is crucial for insulin exocytosis (Henquin, 2011; Rorsman et al., 2012) and modulation of the intracellular S1P level in β‐cells has been shown to affect calcium levels (Hahn et al., 2017). In addition, Lee and colleagues demonstrated that S1P transported in HDL is biologically active and is a major effector of HDL‐mediated increase in intracellular calcium with SRB1 and the S1PR1 and S1PR3 receptors being required (Lee et al., 2017).

Concerning the role of S1P receptors, Japtok et al. showed that pharmacological S1P2 inhibition can reduce insulin secretion independently of HDL (Japtok et al., 2015). We confirmed these results for basal insulin secretion in the absence of HDL, which was decreased in the presence of the S1P2 antagonist, JTE013. However, the pro‐secretory effects induced by HDL were significantly reduced by the S1P1/3 antagonist VPC23019, but not significantly by the S1P2 antagonist, JTE013. The effect of HDL may mainly involve S1P3, which is the most expressed S1P receptor subtype in primary pancreatic beta cells (18 fold higher than S1P1; see Figure 3a).

We were unable to demonstrate any effects of HDL on genes involved in insulin biosynthesis. These results differ from Cochran and colleagues who demonstrated that HDL treatment on MIN6 increase the expression of *Insulin 1*, *Insulin 2*, *Insulin receptor substrate 1* (*Irs1*), *Insulin receptor substrate 2* (*Irs2*) and *Pdx1* (Cochran et al., 2014). The expression of *Insulin* and *Pdx1* remained unchanged in our model. This discrepancy may be explained by our use of primary cultured β‐cells compared to a derived cell line. We also investigated the expression of target genes involved in cell homeostasis and stress‐response. Interestingly, we found no difference between conditions. As our studies do not implicate increased insulin synthesis or improved cell survival as mechanisms in our model of HDL‐induced insulin secretion, it suggests an alternative pathway mediated by S1P.

In conclusion, the present manuscript demonstrates that HDL induces an increase of GSIS by β‐cells independently of an effect on insulin biosynthesis and requires both intra‐ and extra‐cellular sources of S1P. We observed a strong correlation between HDL‐S1P and insulin secretion. We thus highlight the importance of the S1P‐content of HDL in insulin secretion. HDL‐S1P may promote insulin secretion directly, as demonstrated in this current study. Our observations also raise the question of whether HDL‐S1P could be used to evaluate the insulin secretion capacity of HDL particle.

## AUTHOR CONTRIBUTION

MAF, YG, and RWJ contributed to the design of the work and wrote the manuscript. MCBR, AT, JS, FV, RD, YG, and MAF performed experiments, ZP recruited participants and obtained informed consent. MCBR, RD, AT, JS, VS, ZP, JP, NV, RWJ, YG, and MAF participated in the analyses. All authors reviewed and edited the paper.

## Supporting information



Fig S1‐S3

Table S1
