# Supplementary material for: Sphingosine‐1‐phosphate as a key player of insulin secretion induced by high‐density lipoprotein treatment
Source: Physiol Rep. 2021 Mar 26;9(6):e14786. doi: 10.14814/phy2.14786 (PMC7995544; doi:10.14814/phy2.14786)
Supplement: Supplementary file 1 — Fig S1‐S3 [file PHY2-9-e14786-s001.pptx]

## Slide 1
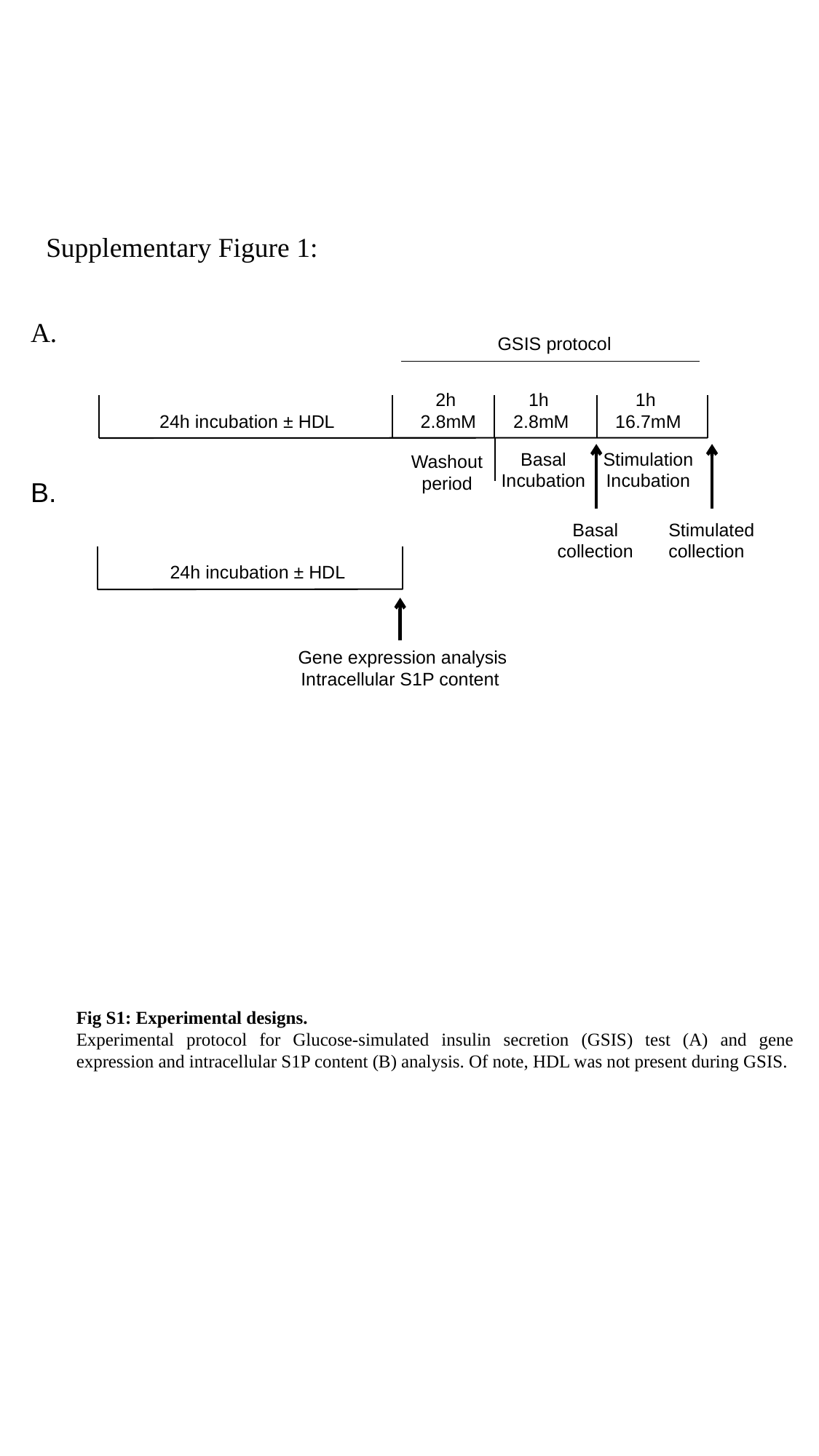

Supplementary Figure 1:
A.
GSIS protocol
2h
2.8mM
1h
2.8mM
1h
16.7mM
24h incubation ± HDL
Basal
Incubation
Stimulation
Incubation
Washout
period
B.
Basal
collection
Stimulated
collection
24h incubation ± HDL
Gene expression analysis
Intracellular S1P content
Fig S1: Experimental designs.
Experimental protocol for Glucose-simulated insulin secretion (GSIS) test (A) and gene expression and intracellular S1P content (B) analysis. Of note, HDL was not present during GSIS.

## Slide 2
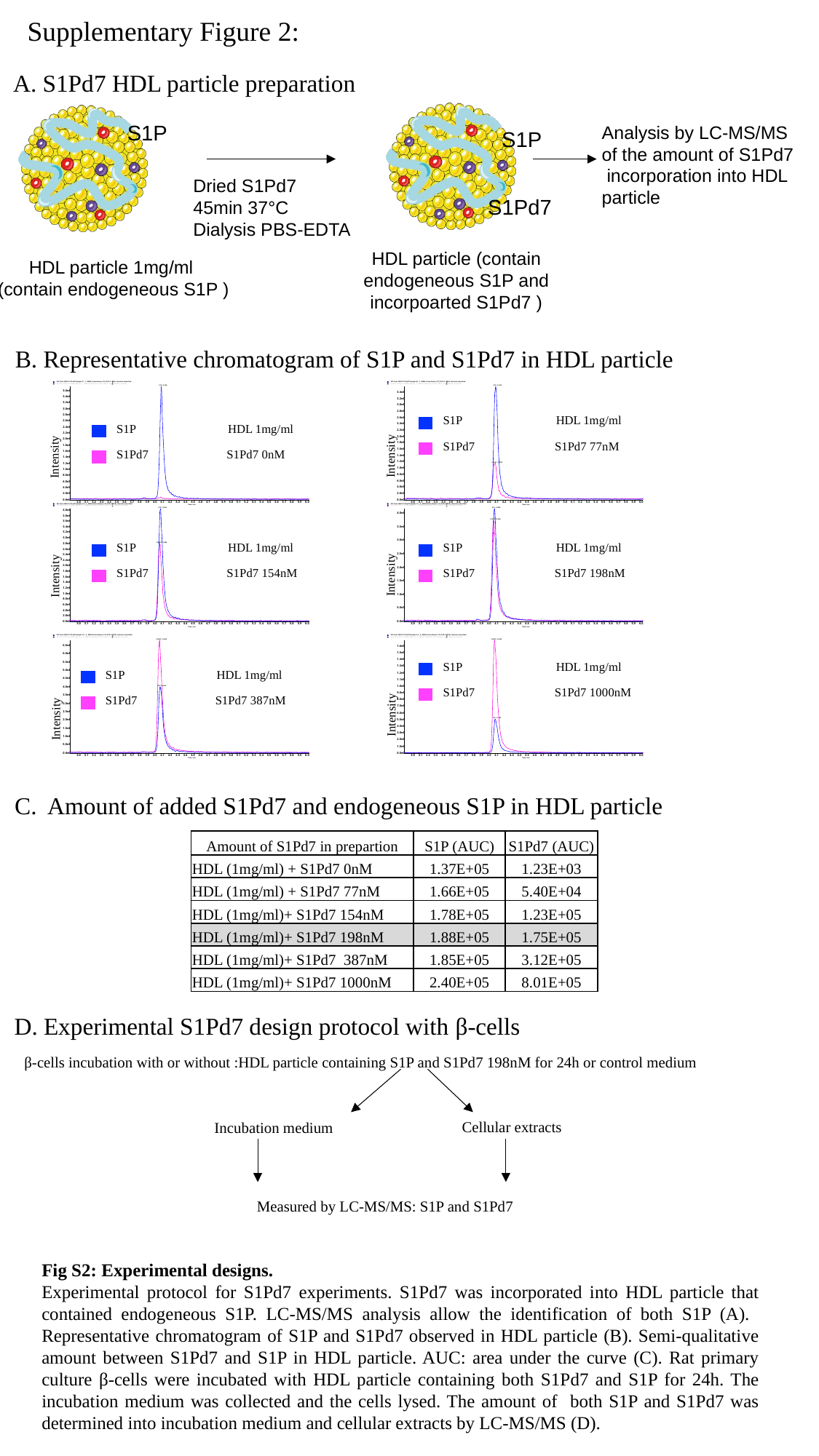

Supplementary Figure 2:
A. S1Pd7 HDL particle preparation
S1P
Analysis by LC-MS/MS
of the amount of S1Pd7
 incorporation into HDL particle
S1P
Dried S1Pd7
45min 37°C
Dialysis PBS-EDTA
S1Pd7
HDL particle (contain endogeneous S1P and incorpoarted S1Pd7 )
HDL particle 1mg/ml
(contain endogeneous S1P )
B. Representative chromatogram of S1P and S1Pd7 in HDL particle
S1P
S1Pd7
HDL 1mg/ml
S1Pd7 77nM
S1P
S1Pd7
HDL 1mg/ml
S1Pd7 0nM
Intensity
Intensity
S1P
S1Pd7
HDL 1mg/ml
S1Pd7 154nM
S1P
S1Pd7
HDL 1mg/ml
S1Pd7 198nM
Intensity
Intensity
S1P
S1Pd7
HDL 1mg/ml
S1Pd7 1000nM
S1P
S1Pd7
HDL 1mg/ml
S1Pd7 387nM
Intensity
Intensity
C. Amount of added S1Pd7 and endogeneous S1P in HDL particle
| Amount of S1Pd7 in prepartion | S1P (AUC) | S1Pd7 (AUC) |
| --- | --- | --- |
| HDL (1mg/ml) + S1Pd7 0nM | 1.37E+05 | 1.23E+03 |
| HDL (1mg/ml) + S1Pd7 77nM | 1.66E+05 | 5.40E+04 |
| HDL (1mg/ml)+ S1Pd7 154nM | 1.78E+05 | 1.23E+05 |
| HDL (1mg/ml)+ S1Pd7 198nM | 1.88E+05 | 1.75E+05 |
| HDL (1mg/ml)+ S1Pd7 387nM | 1.85E+05 | 3.12E+05 |
| HDL (1mg/ml)+ S1Pd7 1000nM | 2.40E+05 | 8.01E+05 |
D. Experimental S1Pd7 design protocol with β-cells
β-cells incubation with or without :HDL particle containing S1P and S1Pd7 198nM for 24h or control medium
Cellular extracts
Incubation medium
Measured by LC-MS/MS: S1P and S1Pd7
Fig S2: Experimental designs.
Experimental protocol for S1Pd7 experiments. S1Pd7 was incorporated into HDL particle that contained endogeneous S1P. LC-MS/MS analysis allow the identification of both S1P (A). Representative chromatogram of S1P and S1Pd7 observed in HDL particle (B). Semi-qualitative amount between S1Pd7 and S1P in HDL particle. AUC: area under the curve (C). Rat primary culture β-cells were incubated with HDL particle containing both S1Pd7 and S1P for 24h. The incubation medium was collected and the cells lysed. The amount of both S1P and S1Pd7 was determined into incubation medium and cellular extracts by LC-MS/MS (D).

## Slide 3
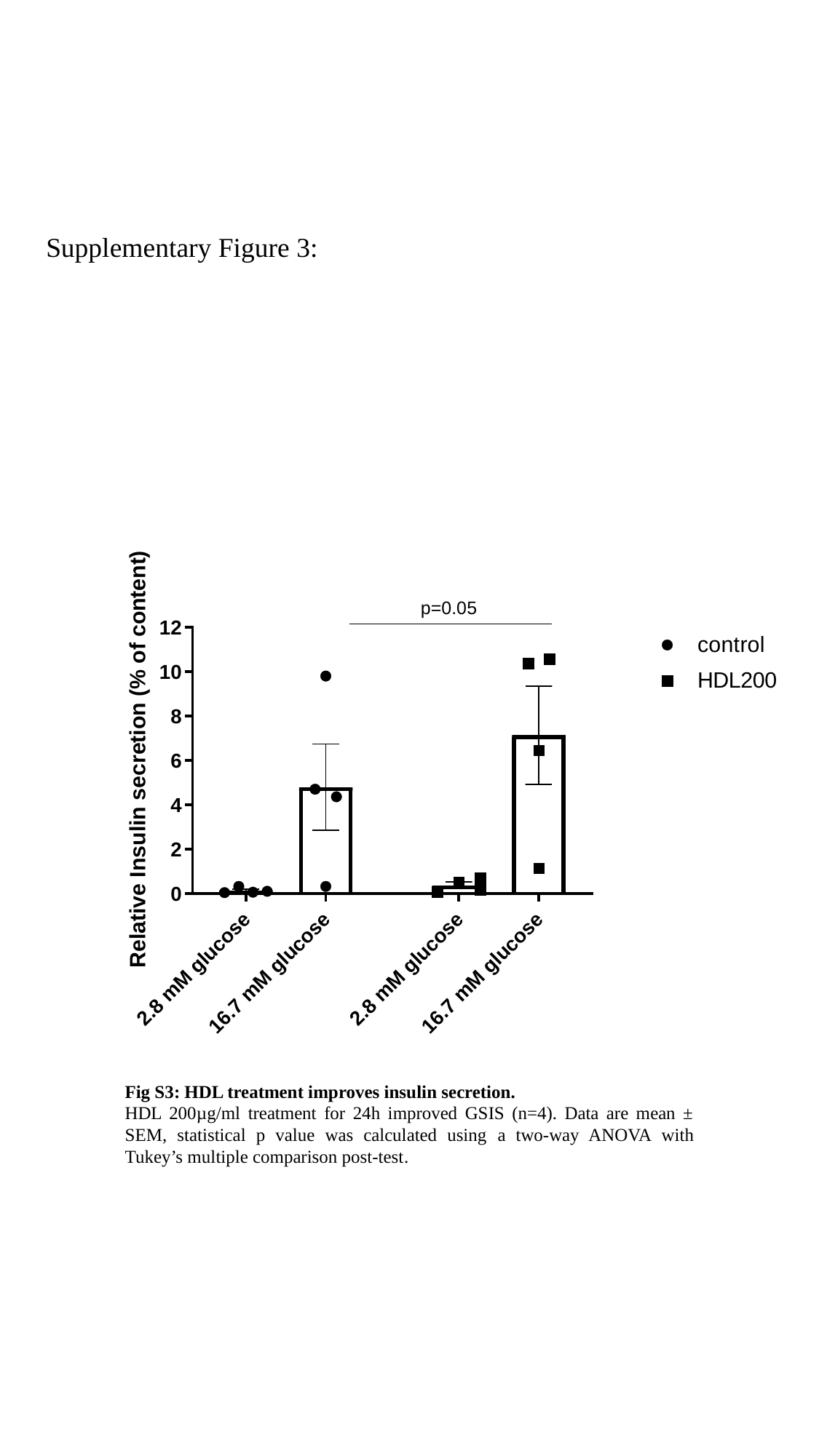

Supplementary Figure 3:
p=0.05
Fig S3: HDL treatment improves insulin secretion.
HDL 200µg/ml treatment for 24h improved GSIS (n=4). Data are mean ± SEM, statistical p value was calculated using a two-way ANOVA with Tukey’s multiple comparison post-test.
