# Supplementary material for: Sphingosine‐1‐phosphate as a key player of insulin secretion induced by high‐density lipoprotein treatment
Source: Physiol Rep. 2021 Mar 26;9(6):e14786. doi: 10.14814/phy2.14786 (PMC7995544; doi:10.14814/phy2.14786)
Supplement: Supplementary file 2 — Table S1 [file PHY2-9-e14786-s002.docx]

**Supplementary Table 1**

Primers table

| Name of gene | Primers Forward 5’-3’ | Primers Reverse 5’-3’ |
| --- | --- | --- |
|  |  |  |
|  |  |  |
|  |  |  |
| *Insulin* | tcttctacacacccaagtcc | tgcagcactgatccacaatg |
| *MafA* | cattctggagagcgagaagtg | tttctccttgtacaggtcccg |
| *Pdx1* | ggatgaaatccaccaaagctc | gtgtaggcagtacgggtcctc |
| *Pax6* | ctagcgaaaagcaacagatgg | cttcctgttgctggcagcc |
| *Pc1* | gggctgaacaacagtatgaaaaag | catgaaggtccagcttgggc |
| *Pc2* | ggtactgaccctcgaaacaaatgcatgtg | ggaggtcatgttgatgttcaggtctcc |
| *Nkx6.1* | aaaacacaccagacccac | gcttctttctccactt |
| *Tpx2* | gggacaaagaacgccagttgc | ggtgtcaaaatggggcacagg |
| *Ki67* | gcccgaccctacaaaatgctg | cactcatctgctgctgcttctcc |
| *Ddit3* | ggtcctgtcctcagatgaaattgg | cgcagggtcaagagtagtgaaggt |
| *Bbc3* | atggcggacgacctcaacg | ggggaggagtcccatgaagag |
| *HspA5* | cacttggtattgaaactgtgggagg | ccagtcagatcaaatgtacccagaag |
| *Top2A* | tggcttctaggaatgcttggtgc | ccttctgctgagcttctttccagg |
| *S1p1* | ggatctatccttggtgaggactc | ccagcaggcaatgaagacactc |
| *S1p2* | actcccttgcagtggtttgc | cgtagagcttgaccttggcg |
| *S1p3* | tcatcagcatcttcacagccattc | atggatctctcggagttgtggtt |
| *S1p4*  *S1p5*  *Rps9* | cgctgggtgtactactgcc  ttactggatgtcgcgtgc  ctccggaacaaacgtgaggt | ccagtgcacaggtgacagct  ggtctcggttggtgaaggtg  tccagcttcatcttgccctc |
